# A biomechanical, micro-computertomographic and histological analysis of the influence of diclofenac and prednisolone on fracture healing in vivo

**DOI:** 10.1186/s12891-016-1241-2

**Published:** 2016-09-05

**Authors:** Oliver Bissinger, Kilian Kreutzer, Carolin Götz, Alexander Hapfelmeier, Christoph Pautke, Stephan Vogt, Gabriele Wexel, Klaus-Dietrich Wolff, Thomas Tischer, Peter Michael Prodinger

**Affiliations:** 1 Department of Oral and Maxillofacial Surgery, Klinikum rechts der Isar der Technischen Universität München, Ismaninger Str. 22, 81675 Munich, Germany; 2 Institute of Medical Statistics and Epidemiology, Klinikum rechts der Isar der Technischen Universität München, Ismaninger Str. 22, 81675 Munich, Germany; 3 Department of Orthopaedics and Orthopaedic Sports Medicine, Klinikum rechts der Isar der Technischen Universität München, Ismaninger Str. 22, 81675 Munich, Germany; 4 Department of Orthopaedic Sports Medicine, Hessing Stiftung Augsburg, Hessingstr. 17, 86199 Augsburg, Germany; 5 Department of Orthopaedic Surgery, University of Rostock, Doberanerstr. 142, 18057 Rostock, Germany

**Keywords:** Fracture healing, Bone regeneration, Bone remodelling, Micro-CT (μCT), Histology, Biomechanics, Bone volume (BV), Tissue mineral density (TMD)

## Abstract

**Background:**

Non-steroidal anti-inflammatory drugs (NSAIDs) have long been suspected of negatively affecting fracture healing, although numerous disputes still exist and little data are available regarding diclofenac. Glucocorticoids interfere in this process over a similar and even broader mechanism of action. As many previously conducted studies evaluated either morphological changes or biomechanical properties of treated bones, the conjunction of both structural measures is completely missing. Therefore, it was our aim to evaluate the effects of diclofenac and prednisolone on the fracture callus biomechanically, morphologically and by 3-dimensional (3D) microstructural analysis.

**Methods:**

Femura of diclofenac-, prednisolone- or placebo-treated rats were pinned and a closed transverse fracture was generated. After 21 days, biomechanics, micro-CT (μCT) and histology were examined.

**Results:**

The diclofenac group showed significantly impaired fracture healing compared with the control group by biomechanics and μCT (e.g. stiffness: 57.31 ± 31.11 N/mm vs. 122.44 ± 81.16 N/mm, *p* = 0.030; callus volume: 47.05 ± 15.67 mm3 vs. 67.19 ± 14.90 mm3, *p* = 0.037, trabecular thickness: 0.0937 mm ± 0.003 vs. 0.0983 mm ± 0.003, *p* = 0.023), as confirmed by histology. Biomechanics of the prednisolone group showed obviously lower absolute values than the control group. These alterations were confirmed in conjunction with μCT and histology.

**Conclusions:**

The inhibiting effects of both substances were not only mediated by absolute parameters (e.g. breaking load, BV), but we have shown, for the first time, that additional changes occurred in the microstructural bony network. Especially in patients at risk for delayed bone healing (arteriosclerosis, diabetes mellitus, smoking), the administration of these drugs should be weighed carefully.

**Electronic supplementary material:**

The online version of this article (doi:10.1186/s12891-016-1241-2) contains supplementary material, which is available to authorized users.

## Background

In clinical practice, several medications offering pain relief, anti-inflammation and a reduction of postoperative swelling are applied after bone surgery or trauma, although their potential side effects on bone healing have not been studied adequately. Non-steroidal anti-inflammatory drugs (NSAIDs) have for some time been suspected to affect fracture healing negatively [1–4] and might even cause bone loss after tooth extraction [5]. However, numerous disputes still exist and limited data are available with regard to diclofenac, which is one of the most often applied substances in clinical use. Through the inhibition of phospholipase A2, glucocorticoids act earlier in the same pathway as NSAIDs. Their possibly more serious consequences after short-term use for humans have been insufficiently examined in this context. They are known to affect bone remodelling [6–8] and fracture healing in animal models after long-term use [9, 10].

Recent molecular biological studies suggest that the early bone healing phase is crucial for the definitive success and stability of the bone [11]. This phase is characterised by an inflammatory reaction of the body with an increase in the secretion of prostaglandins (PGs) by osteoblasts [12].

PGs are hormone-like substances with proinflammatory effect and play a key role in fracture healing. The cyclooxygenase (COX) isoenzymes 1 and 2 (COX-1 and -2) control the production of PGs: COX-2 is specifically involved in the inflammatory response, whereas COX-1 is rather involved universally in various physiological processes, such as platelet aggregation and cytoprotection in the gastrointestinal tract. NSAIDs inhibit unspecifically both the activity of COX-1 and -2 and effectively reduce pain and inflammation [13].

In vitro, diclofenac acts negatively on osteoblasts at early stages and appears to inhibit their function. This effect is seen even at relatively low concentrations corresponding to those commonly reached in vivo and might possibly lead to a delay of bone healing [11, 13]. Additionally, NSAIDs are used therapeutically to reduce heterotopic ossifications after elective joint-replacement or fractures, strongly suggesting their potential to inhibit or negatively affect endochondral ossification mechanisms [4]. Nevertheless, within the few animal studies previously carried out, the reported influence of diclofenac on bone healing is controversial and, thus, unclear [2, 13–15].

Physiological humane serum levels of glucocorticoids have shown a maximum stimulatory effect on osteoblasts in vitro. However, increasing the dose in vitro to supraphysiological doses leads to a decreased ability of osteoblasts to differentiate [6]. Corticosteroid treatment is commonly used in inflammatory and rheumatological diseases and to reduce postoperative pain and prolonged soft tissue swelling after elective surgery or accidental trauma [16]. Unlike NSAIDs, which do not interfere until the conversion of arachidonic acid to PGs in the biosynthetic pathway, glucocorticoids inhibit the production of arachidonic acid and thus interfere at an earlier point than NSAIDs in this pathway [17]. Arachidonic acid is the basis for the production of both PGs and leukotrienes. In addition to the PGs, the latter play a crucial role in the inflammation response of the body. Consequently, glucocorticoids might lead to a (more) serious delay of bone healing [7, 18, 19].

Both drugs are often administered in clinical use briefly after surgery or trauma. Therefore, the combined results of our study should allow to reduce uncertainties of former studies concerning the impact of medication on the early stage of fracture healing and should enrich our knowledge of the way in which these drugs act. Unlike precedent work, our evaluation was performed functionally, microstructurally and morphologically via the combination of biomechanics, micro-CT (μCT) and histology.

Summarised, our main objectives were to examine whether the tested substances (1) show the potential to reduce the load bearing capacity of the fracture callus of unstable mid-femural fractures expressed by a reduced breaking load in the three-point bending and (2) whether microstructural changes of the newly formed callus are evident by μCT and histology.

## Methods

### Animal model

Adult male Wistar rats (*n* = 63, aged 16 weeks, mean weight ± SD: 500 g ± 50 g) were purchased from Charles River Laboratories (Sulzfeld, Germany) and acclimatised for at least 2 weeks prior to experimentation. The animals were singly fed, housed at 23–25 °C (humidity: 55 ± 5 %) with a 12-h light/dark cycle and allowed free access to water and standard laboratory pellets.

Rats were randomised and allocated to the 3 different arms (Diclofenac, Prednisolone or Control) each consisting of 2 groups (Group A: biomechanical testing: total of 33 rats; 11 animals for diclofenac, 11 for prednisolone and 11 for controls; Group B: histology and μCT: total of 21 rats; 7 animals for diclofenac, 7 for prednisolone and 7 for controls).

Prior to the operation, the rats were anaesthetised by intramuscular (i.m.) injection of Medetomidine (Medetomin, 0.15 mg/kg, Dechra Veterinary Products, ‘s-Hertogenbosch, Netherlands), Midazolam (Midazolam, 2 mg/kg, Hexal AG, Germany) and Fentanyl (Fentadon, 5 μg/kg, Dechra Veterinary Products, ‘s-Hertogenbosch, Netherlands). A Kirschner wire (K-wire; 1.0 mm) was inserted into the medullary canal of the right femur in an antegrade manner [20, 21] followed by a closed mid-diaphyseal fracture. The procedure was performed as a modification of the method first described by Bonnarens and Einhorn in 1984 [22].

To end the anaesthesia after the operation, an antidote combination was given subcutaneously (s.c.), composed of Atipamezole (Antisedan, 0.75 mg/kg, Orion Corporation, Espoo, Finland), Flumazenil (Flumazenil, 0.2 mg/kg, Hexal AG) and Naloxone hydrochloride (Naloxone, 0.12 mg/kg, Braun AG, Germany). During the postoperative period, pain relief was performed by the subcutaneous administration of buprenorphine twice a day (Buprenodale, 0.05 mg/kg, Dechra Veterinary Products) and the animals were able to put weight on the leg immediately.

Depending on the group, daily subcutaneous administration of diclofenac or prednisolone was performed. The control animals received only sodium chloride solution subcutaneously. Drugs were administrated in the following dosages:

- Diclofenac (Voltaren-Resinat, Novartis GmbH): 5 mg/kg BW per day s.c.
- Prednisolon (Solu-Decortin H, Merck KGaA): 0,5 mg/kg BW per day s.c.

Under anaesthesia with isoflurane, blood was taken 2–3 h after the administration of the substances on day 5 from the venous angle to check the serum levels of the medication.

Before the rats were sacrificed on day 21 by an overdose of Narcoren (sodium pentobarbital 80 mg/kg BW), they were anaesthetised in a plastic box by inhalation of isoflurane and, once more, blood was taken by puncture of the heart to determine the serum levels of each drug. Plain X-ray controls (anterior-posterior and lateral view) were performed via c-arm (Siemens, Erlangen, Germany) after intramedullary pinning, following the fracture (both intraoperatively) and post mortem.

Depending on the group allocation, the bones were fresh-frozen and stored at −20 °C (Biomechanical group) or fixed in 100 % methanol (histology/μCT) and stored at 4 °C. Analyses were carried out, after pin removal, either by μCT or by biomechanics. All researchers involved in this study were blinded throughout the evaluations.

### Biomechanics

**Fig. 1:**
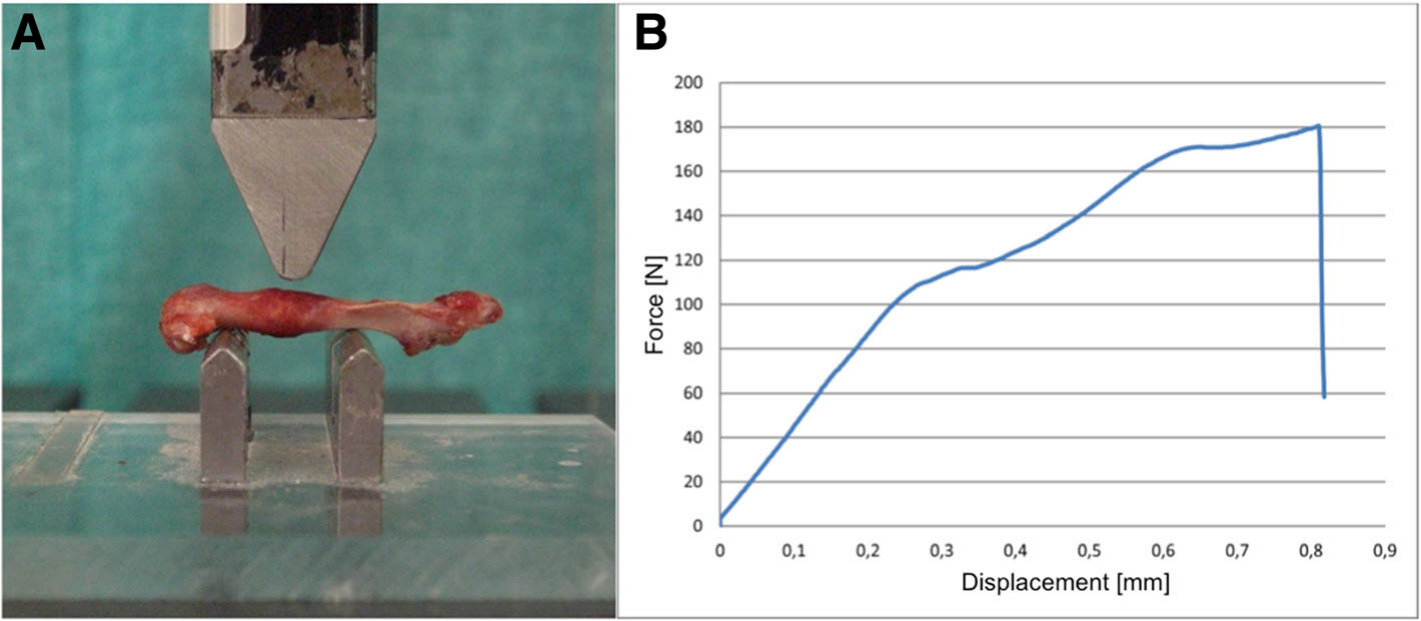
Biomechanical testing. **a** Three-point bending by using a material testing machine. **b** Breaking load (N) and stiffness (N/mm) were determined from the load-displacement diagram (highest point or regression of the curve)

Three-point bending was performed by using a Wolpert TZZ 707/386 material test machine (Istron Wolpert GmbH, Darmstadt, Germany, Fig. 1a). According to Turner et al., the distance between the bearing and loading bars for each rat femur was 15 mm for 3-point bending [23]. The femurs were placed horizontally with the anterior surface upwards.

Bending load was applied constantly with a displacement rate of 5 mm/min and directed vertically to the mid-shaft of the femur until failure (breaking load). The termination criterion was defined as a reduction in the force of > 50 N, whereas the failure criterion was defined as a reduction in the force of 80 %. The breaking load (N) and stiffness (N/mm) were determined from the load-displacement diagram (highest point or regression of the curve) (Fig. 1b) by using the test program Test&Motion (DOLI Elektronik GmbH, München, Germany).

Failure load and stiffness were collected for each femur of the biomechanical group. Absolute and relative values [failure load and stiffness of the experimental side (*n* = 30) in relation to the intact contralateral bone (*n* = 30) as a percentage (%) of the intact load or stiffness] were determined.

### Micro CT

**Fig. 2:**
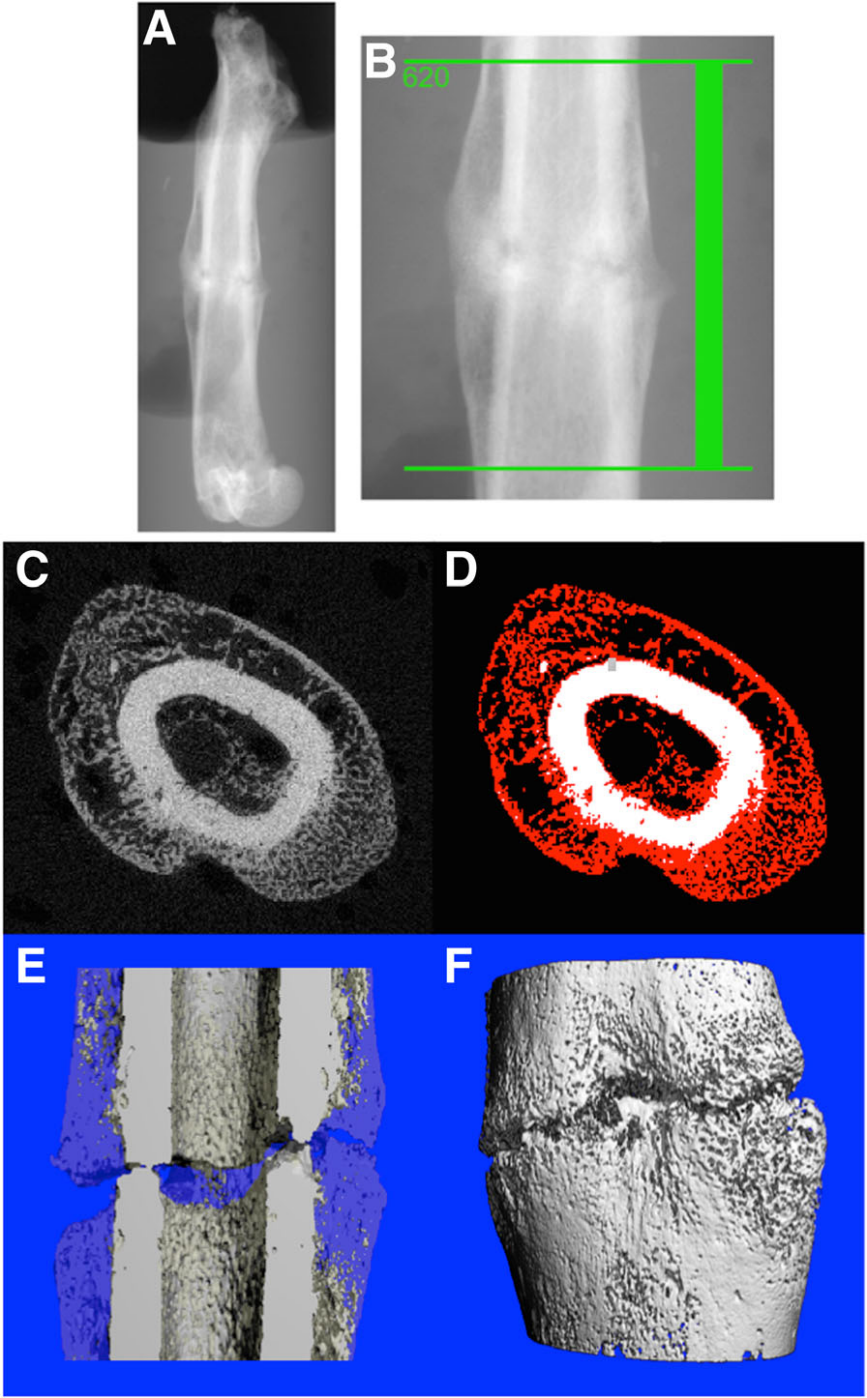
Scout view (**a**) and determination of the scanning area within two reference lines (**b**). 2D axial μCT (**c**) *grey* value image of original cortical bone, callus and air/bone marrow and (**d**) corresponding full automatically segmented image (callus = red); 3D coronar μCT half-sliced (**e**) and overview image (**f**) (Two Thresholding Procedure, callus blue and semitransparent and cortical bone grey)

The femura were scanned by using an isotropic voxel size of 10 μm (55 kVp, 145 μA; μCT 40, Scanco Medical, Brüttisellen, Switzerland). The integration time was set at 200 ms. Images were reconstructed with 2048 × 2048 pixels per cross section. Before the measurement, a scout view was obtained and the scanning area of 620 μm (slice increment: 10 μm) covering both sides of the fracture gap (each 3.1 mm) was determined within two reference lines (Fig. 2a and b).

Thresholds were determined visually by two independent examiners (based on histograms) to separate original cortical bone from callus, marrow and solution [24]. Differences in the brightness of the pixels were evident (Fig. 2c and d). A constrained 3D Gaussian filter was used to suppress partly the noise in the volumes. The grey-scale images were segmented by using the same parameters for callus [sigma (0.8), support (1) and threshold (150)] and original cortical bone [sigma (1.5), support (3) and threshold (370)]. After reconstruction of the data, the analysis of the micro-structural parameters was performed on the basis of the selected volume of interest (VOI) to obtain the 3D evaluation. A standard convolution-backprojection procedure with a Shepp and Logan filter was used to reconstruct 3D CT images (Fig. 2e and f). All image processing steps were conducted automatically by using Image Processing Language (IPL, Institute for Biomedical Engineering, ETH and University of Zürich). The following non-volume-depending parameters (as metric characteristics) were determined: the bone volume (BV, mm^3^), tissue mineral density (TMD, mg HA/cm^3^) and bone mineral content (BMC, defined as the callus BV multiplied by TMD, mg) [25] and the structure model index (SMI, dimensionless), degree of anisotropy (DA, dimensionless), bone surface (BS, mm3) and trabecular thickness (Tb. Th., mm) as structural parameters.

### Histological analysis

After μCT analysis, the specimens were dehydrated in a graded series of ethanol (from 70 to 100 % [v/v]) and acetone and were then embedded in methyl methacrylate (MMA). After polymerisation, undecalcified sections of the MMA-embedded samples of 100 ± 20 μm in thickness were prepared by using a sawing microtome (Leica, Wetzlar, Germany) technique. Sections were cut coronally through each sample. Selected specimens were additionally ground (70 μm) and polished (Schleifsystem 400 CS, Exakt, Norderstedt, Germany). For histological analysis, the sections were surface-stained as described by Laczkó and Lévai (LL) [26]. For overview images, a Wild® Macroscope M3Z (Wild, Heerbrugg, Switzerland) was used in motion function and analysis was performed via bright-field microscopy (Axiophot 2; Zeiss, Jena, Germany). Detailed images were digitised with a microscope (Nikon Eclipse 50i; Nikon, Düsseldorf, Germany) and video camera (AxioCam HRc; Zeiss, Jena, Germany; magnification 10x). Semi-quantitative analysis was carried out by using an image analysis system (Axiovision 4.8, Zeiss, Jena, Germany) modified from [14, 20]. In detail, the area of the fracture gap of two central sections was examined. We semi-quantitatively evaluated whether, in the respective area cartilage, connective tissue or newly formed bone was present. This was described as a percentage of all the samples of each group with the respective tissue. Furthermore, we investigated whether bony bridging of the fracture gap appeared. This was reported as a percentage of all the samples of each group.

### Statistical analysis

A power analysis was used to determine the number of animals needed for the biomechanical workup [27]. A two group t-test with a 5 % two-sided significance level has 80 % power to detect an effect size of 1.38 (or 1.26) when the sample sizes in two groups are 8 (or 11) and 11, respectively (nQuery Advisor 7.0). With regard to μCT and histology, a minimal sample size of 6 animals was chosen for exploratory investigations and the computation of descriptive statistics with no need for a power calculation.

Statistical analysis was carried out by using R 3.1.0 (The R Foundation for Statistical Computing, Vienna, Austria) and GraphPad Prism Version 4.00 (GraphPad Prism Software® San Diego, USA). Differences between the treatment groups were assessed by unpaired two-sided Student’s t-tests. All tests were conducted on exploratory 5 % significance levels. The distribution of quantitative data was imaged as mean values ± standard deviation (mean ± SD).

## Results

### Inclusions and exclusions

All in all, 49 of the animals could be included in the study. No infection, weight loss ≥ 5 % or severe swelling was observed. 14 of 63 (22 %) had to be excluded because of various complications (no fracture, comminuted fracture or wrong localisation of the fracture).

### Serumlevels

Diclofenac blood levels were determined to be within the target range (0.1–2.5 μg/ml; Medizinisches Versorgungszentrum Dr. Eberhard & Partner, Dortmund; first blood draw: 1.34 μg/ml; second blood draw: 2.33 μg/ml, averages).

Prednisolone blood levels were also determined to lie within the target range (30–400 ng/ml) detectable (first blood draw: 94.05 ng/ml; second blood draw: 40.01 ng/ml, averages).

### Biomechanics

Thirty animals (60 femura) were eligible for biomechanical testing.

**Fig. 3:**
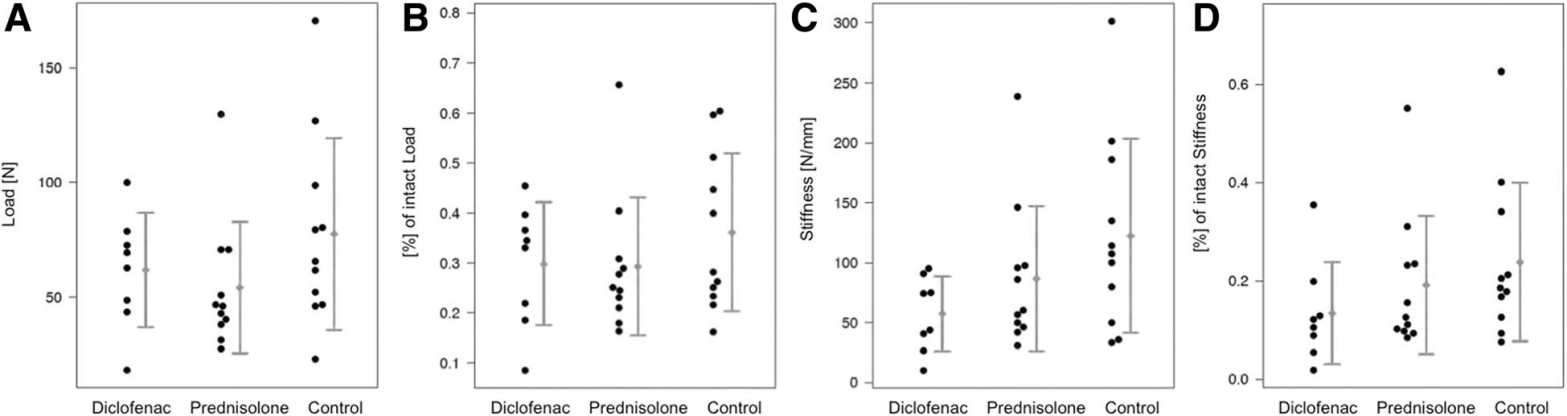
Biomechanical values as means ± SD and dotplot of (**a**) breaking load and (**b**) % of intact load, (**c**) stiffness and (**d**) % of intact stiffness. Mean differences and significances are shown in Table 1

Regarding the breaking load, the Diclofenac and prednisolone groups showed obviously lower values than the control. In detail, the breaking load of the experimental sides exhibited 77.65 N ± 41.82 N for the controls, 61.97 N ± 24.91 N for the diclofenac group and 54.30 N ± 28.68 N for the prednisolone group (Table 1, Fig. 3a). The percentage of the intact load revealed 0.36 ± 0.16 for the controls, 0.30 ± 0.12 for the diclofenac group and 0.29 ± 0.14 for the prednisolone group (Table 1, Fig. 3b).

**Table 1:** Biomechanical parameters

		Mean ± SD		Mean difference (95 %-CI)	p-value
Breaking load	D	61.97 ± 24.91	D vs P	7.67 (-18.45 – 33.79)	0.543
	P	54.30 ± 28.68	D vs K	-15.68 (-48.20 – 16.84)	0.323
	K	77.65 ± 41.82	P vs K	23.35 (-8.81 – 55.51)	0.144
% of intact load	D	0.30 ± 0.12	D vs P	0.01 (-0.12 – 0.13)	0.934
	P	0.29 ± 0.14	D vs K	-0.06 (-0.20 – 0.07)	0.345
	K	0.36 ± 0.16	P vs K	0.07 (-0.06 – 0.20)	0.297
Stiffness	D	57.31 ± 31.11	D vs P	-29.32 (-74.53 – 15.89)	0.188
	P	86.63 ± 60.45	D vs K	-65.13 (-122.81 – -7.45)	0.030*
	K	122.44 ± 81.16	P vs K	35.81 (-28.18 – 99.79)	0.256
% of intact stiff-ness	D	0.13 ± 0.10	D vs P	-0.06 (-0.18 – 0.06)	0.321
	P	0.19 ± 0.14	D vs K	-0.10 (-0.23 – 0.03)	0.107
	K	0.24 ± 0.16	P vs K	0.05 (-0.09 – 0.18)	0.481

D = diclofenac (*n* = 8), P = prednisolone (*n* = 11), K = control (*n* = 11). Entries marked with *represent significant differences between therapy groups

Regarding the stiffness, significant differences were generated (*p* = 0.030) between the control and diclofenac groups. In detail, the stiffness of experimental bones revealed 122.44 ± 81.16 N/mm for the controls, 57.31 ± 31.11 N/mm for the diclofenac group and 86.63 ± 60.45 N/mm for the prednisolone group (Table 1, Fig. 3c). The percentage of the intact stiffness exhibited 0.24 ± 0.16 in the control group, 0.13 ± 0.10 in the diclofenac group and 0.19 ± 0.14 in the prednisolone group (Table 1, Fig. 3d).

### μCT and histological examination

Nineteen animals were eligible for μCT and histology.

**Fig. 4:**
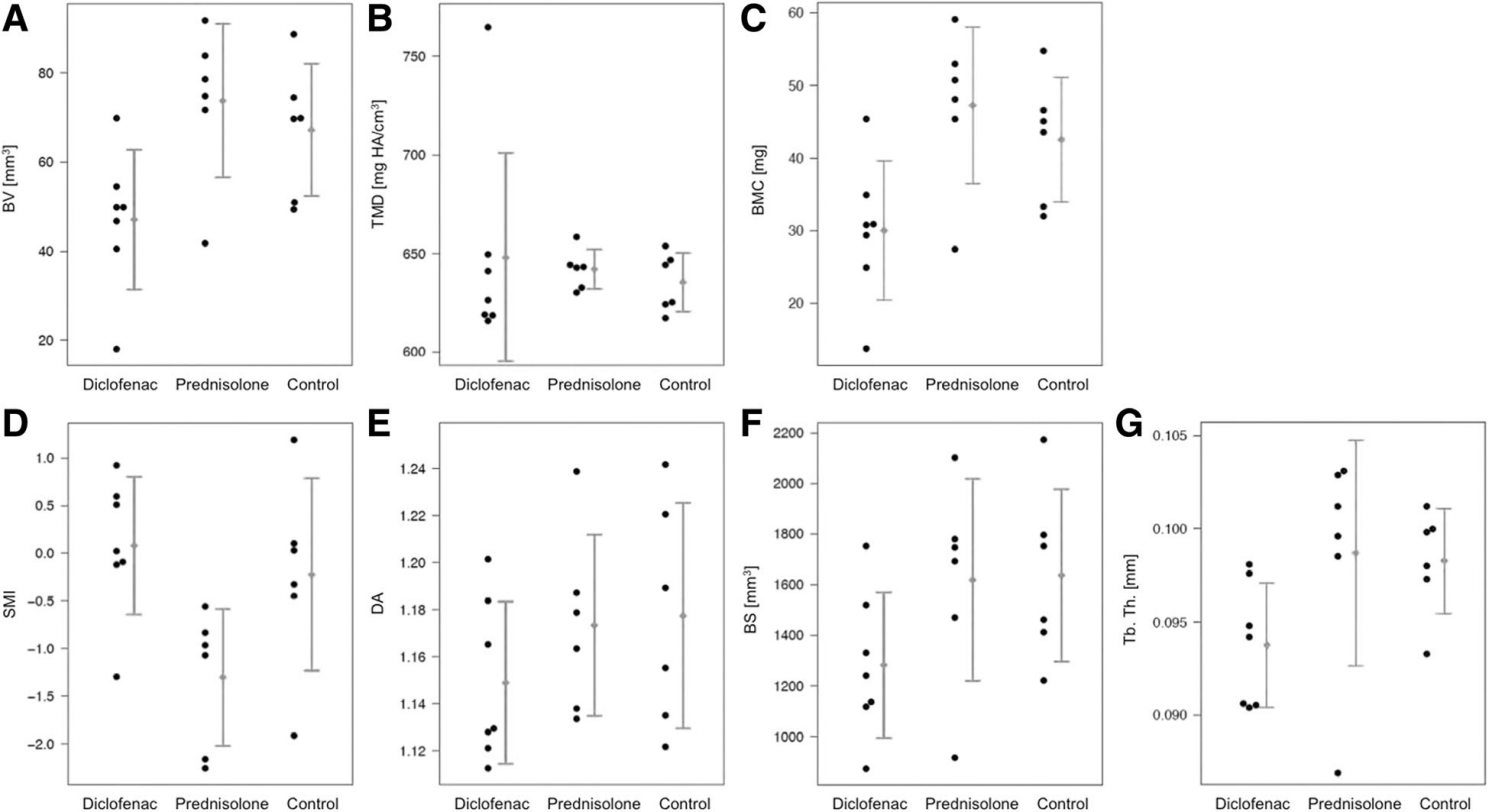
μCT values as means ± SD and dotplot of (**a**) BV, (**b**) TMD, (**c**) BMC, (**d**) SMI, (**e**) DA, (**f**) BS and (**g**) Tb. Th.. Mean differences and significances are displayed in Table 2

BV was the most objective parameter with which to assess the effective, i.e. absolute, callus volume (peri- and endosteal) without the original cortical bone. The control group exhibited a significant higher BV than the diclofenac group (67.19 ± 14.90 vs. 47.05 ± 15.67, *p* = 0.037). Prednisolone generated the highest callus volume (73.79 ± 17.23), which was significantly higher than that of the diclofenac group (*p* = 0.015, Table 2, Fig. 4a). With respect to the TMD, very homogeneous results were evident throughout the groups, except for the SD of the diclofenac group: the latter showed an outlier (52.91) that strongly influenced the mean and SD (SD control = 14.81 and SD prednisolone = 9.99) (Table 2 and Fig. 4b). BMC represents density x volume. It exhibited analogous significances and trends in values to those for BV (Table 2 and Fig. 4c).

**Table 2:** μCT callus parameters

		Mean ± SD		Mean difference (95 %-CI)	p-value
BV	D	47.05 ± 15.67	D vs P	-26.74 (-47.15 – -6.33)	0.015*
	P	73.79 ± 17.23	D vs K	-20.14 (-38.86 – -1.42)	0.037*
	K	67.19 ± 14.90	P vs K	6.60 (-14.17 – 27.38)	0.494
TMD	D	648.10 ± 52.91	D vs P	6.04 (-42.99 – 55.08)	0.776
	P	642.06 ± 9.99	D vs K	12.62 (-36.68 – 61.92)	0.565
	K	635.48 ± 14.81	P vs K	6.58 (-9.98 – 23.14)	0.391
BMC	D	30.01 ± 9.58	D vs P	-17.27 (-29.92 – -4.61)	0.012*
	P	47.28 ± 10.76	D vs K	-12.52 (-23.63 – -1.41)	0.031*
	K	42.54 ± 8.61	P vs K	4.75 (-7.87 – 17.36)	0.420
SMI	D	0.08 ± 0.72	D vs P	1.38 (0.50 – 2.27)	0.006*
	P	-1.30 ± 0.72	D vs K	0.30 (-0.81 – 1.42)	0.553
	K	-0.22 ± 1.01	P vs K	-1.08 (-2.22 – 0.06)	0.062
DA	D	1.15 ± 0.03	D vs P	-0.02 (-0.07 – 0.02)	0.258
	P	1.17 ± 0.04	D vs K	-0.03 (-0.08 – 0.02)	0.258
	K	1.18 ± 0.05	P vs K	0.00 (-0.06 – 0.05)	0.877
BS	D	1282.18 ± 288.17	D vs P	-336.62 (-779.83 – 106.59)	0.120
	P	1618.80 ± 398.72	D vs K	-354.66 (-748.85 – 39.54)	0.073
	K	1636.83 ± 340.78	P vs K	-18.04 (-496.72 – 460.65)	0.935
Tb. Th.	D	0.094 ± 0.003	D vs P	-0.005 (-0.011 – 0.001)	0.114
	P	0.099 ± 0.006	D vs K	-0.005 (-0.008 – -0.001)	0.023*
	K	0.098 ± 0.003	P vs K	0.000 (-0.006 – 0.007)	0.878

D = diclofenac (*n* = 7), P = prednisolone (*n* = 6), K = control (*n* = 6). Entries marked with *represent significant differences between therapy groups

Structural parameters uniformly describe and quantify the microarchitecture of specimens (Table 2).

The SMI of the control group showed no relevant difference from the diclofenac group (*p* = 0.553). Prednisolone showed the smallest mean (-1.30 ± 0.72). This was significantly reduced compared with that of the diclofenac group (0.08 ± 0.72, *p* = 0.006) (Table 2 and Fig. 4d). With respect to the mean and SD of DA, all three groups here were very homogeneous without relevant differences (Table 2 and Fig. 4e). The mean of BS in the control group (1636.83 ± 340.78) was considerably higher than that in the diclofenac group (1282.18 ± 288.17, *p* = 0.073). In contrast, the mean of BS in the control group was only slightly higher than that of the prednisolone group (1618.80 ± 398.72, *p* = 0.935, Table 2 and Fig. 4f). With respect to Tb. Th., a significant difference (*p* = 0.023) was seen between the control group (mean = 0.0983 ± 0.003) and the diclofenac group (mean = 0.0937 ± 0.003) (Table 2 and Fig. 4g).

**Fig. 5:**
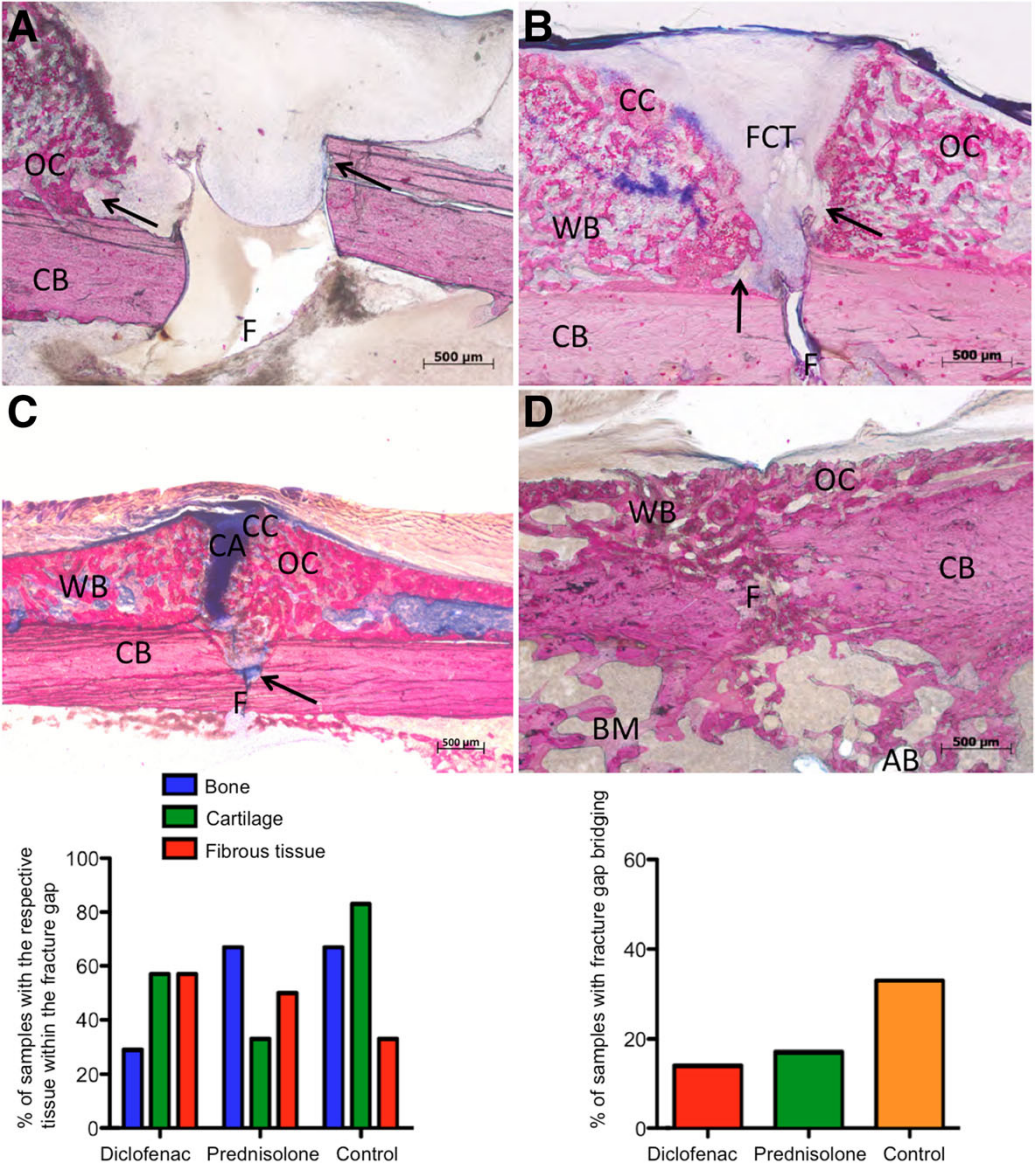
Representative histological images of fracture sites of the diclofenac (**a** and **b**), prednisolone (**c**) and control (**d**) groups. Each section is oriented with the cortical bone on the bottom, periosteal fracture callus on the top and fracture site in the middle. Magnification 40 × (**a**, **b**, **d**) or 25 × (**c**). Scale bar: 500 μm. *Left diagram*: % of samples with the respective tissue within the fracture gap; *right diagram*: % of samples with bony fracture gap bridging. The diclofenac-treated rats often developed areas of high bone resorption (denoted by *arrows*) at the CB, the F and the periosteal callus junction. A shows a gap without any signs of a union. Note the lack of CA. B shows a fibrous bone union. In the prednisolone group (**c**), osteocondral bone union and active new bone formations were detected indicating that endochondral ossification had occurred, although the periosteal OC did not bridge the gap. Generally, calluses contained minor amounts of cartilage, but in half of the samples, residual FCT was still detectetable (*left diagram*). D shows a sample with OC only and complete union with trabecular structure (*right diagram*). The normal fatty BM was replaced by bone, the AB seen in D is an artifact of the embedding procedure. Staining: LL (bone: red, cartilage: blue; fibrous connective tissue: pale blue to grey/white). CB = original cortical bone, OC = osseus callus, CA = cartilage, CC = calcified cartilage, WB = woven bone, FCT = fibrous connective tissue, F = fracture site, AB = air bubble, BM = bone marrow, arrow = zone of resorption

Histological findings further supported the radiographic evidence. The control group showed bony callus in the area of the fracture gap analogous to the prednisolone group in 67 % of the samples. In contrast, the diclofenac group showed bony callus in only 29 % of the samples in the area of the fracture gap (Fig. 5 left diagram). Additionally, we investigated cartilage and fibrous connective tissue. In the prednisolone group, the least cartilage was seen within the fracture gap, whereas 50 % of the samples still showed fibrous connective tissue. Both the control group and the diclofenac group generated relatively more cartilage than bone. Fibrous connective tissue as poor-quality tissue was detected least frequently within the fracture gap of the femura of the control group (33 %). In the diclofenac group, in which occasionally diastases of the fracture gap were observed (Fig. 5a), fibrous connective tissue (Fig. 5b) was detected the most, at 57 %. Bone resorption areas were detectable at the cortical bone, the fracture site and the periosteal callus junction (Fig. 5a and b). Furthermore, a (negative) ratio of bone/cartilage/connective tissue (29 %/57 %/57 %) was evident in this group (Fig. 5 left diagram). With regard to the bony bridging of the fracture gap (Fig. 5 right diagram), the control group was more successful (33 %) than the prednisolone group (17 %, despite the high callus BV detected via μCT, Fig. 4a) and the diclofenac group, respectively (14 %). Figure 5c shows a representative sample of the prednisolone group with osteocondral bone union and active new bone formation indicating that endochondral ossification had occurred, although the outer periosteal mineralised callus did not bridge the gap, in contrast to that of the control group (Fig. 5d).

**Fig. 6:**
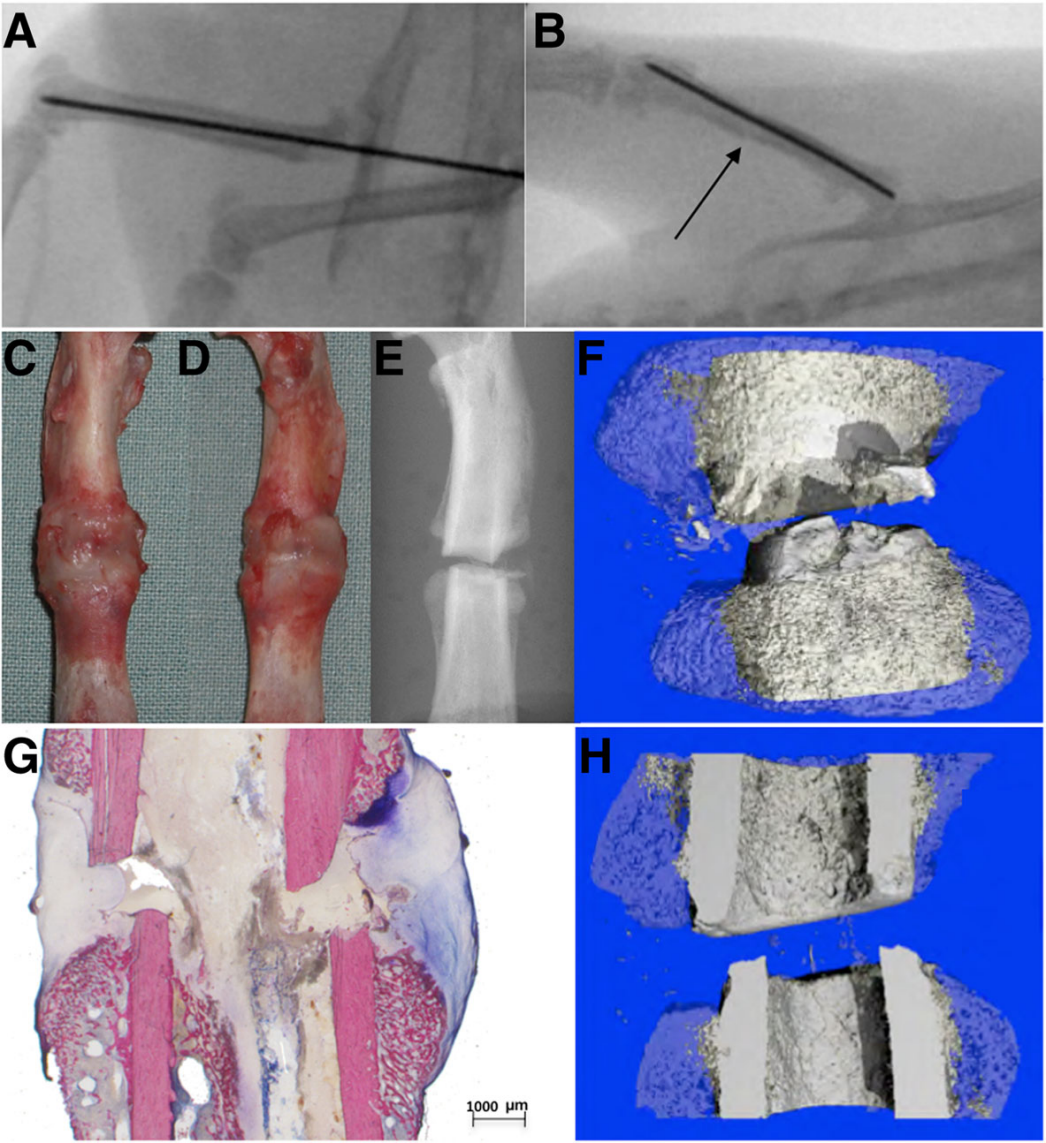
**a** and **b** illustrates intraoperative plain X-ray controls via c-arm after intramedullary pinning and after the fracture. The second row displays (**c** = dorsal, **d** = ventral) macroscpic, (**e**) scout view and (**f**) 3D coronar μCT (callus blue and semitransparent, original cortical bone grey) post mortem controls. **g** shows a histological overview image (10×, details: Fig. 4a) and **h** a corresponding half-sliced 3D coronar μCT reconstruction. All images represent the same specimen of the diclofenac group. Macroscopically, no diastasis of the fracture gap is detectable. However, the tissue type can only be assumed. In contrast, radiologically, a diastasis of the fracture gap and a dislocatio ad latum with wedge-shaped defects is seen. Additionally, histology demonstrates fibrous connective tissue

Figure 6 shows a synopsis of the modalities of group B (histology/μCT).

## Discussion

Numerous disputes and limited data exist on bone healing with regard to the use of diclofenac and prednisolone. Possible causes for these problems are the applied evaluation methods that are no longer considered sufficient by today’s standards. Therefore, our aim was to evaluate the qualitative and quantitative structural properties of the fracture callus in vivo via a combination of biomechanics, micro-CT and histology.

Additional to the expected effects of each drug, the timepoint in our study was chosen based on joint consideration of the time course of rat callus fracture healing after 21 days [10, 28]. At that time, callus formation is not yet finished and stable and significant differences can be detected [29].

The diclofenac dose of 5 mg/kg body weight per day was taken from the literature. Previous publications have shown that a dose of 2.5 times (compared with the human dose of 2 mg/kg body weight) for the rat is necessary to generate human equivalent pharmacological data [2, 15, 30].

The diclofenac group showed significantly lower values for BV and BMC compared with the control and prednisolone groups. Occasionally, we observed diastases of the fracture gap. Histologically, only 29 % of the samples showed mineralised callus in the area of the fracture gap. However, the most common tissue within the gap was fibrous connective tissue (57 %) indicating impairment of fracture healing. Our results are concordant with those of Krischak et al. who have studied this phenomenon histologically. Both the bony percentage in the area of the fracture gap and the fraction of bony bridges were decreased, whereas the proportion of cartilage was increased [14]. Likewise, Tiseo et al. showed a delay of bone remodelling, since the mineralised part of tissue within the callus was low and even decreased over time (by 2-4 weeks) [31]. However, the two studies only evaluated histological samples and did not focus on the 3D structural parameters of bone [11]. Our results substantiate the inhibiting influence of diclofenac on fracture healing. New bone formation seems to be reduced by diclofenac, whereas resorption seems to be increased. Similar density values were determined between the groups. If, in spite of similar TMD values, different SMI values are detected, then the SMI reveals that different trabecular structures are present. Values of zero and negative values represent a more stable plate-like structure, whereas higher values indicate a less stable rod-like structure. In the dioclofenac group, the SMI was the highest (0.08 ± 0.72) and took a positive value only in this group. This indicates the presence of less stable bone in the diclofenac-treated group with a reduced capacity to adapt load [32]. Furthermore, the trabecular thickness was significantly reduced by diclofenac compared with the control group implying a weaker network. Previous studies applied mainly conventional radiological (or peripheral quantitative computed tomography = pQCT), histological and/or biomechanical results without being able to detect 3D trabecular structures [2, 14, 15]. Thus, with regard to our results, not only the reduced amount of bone substantiates the inhibiting influence of diclofenac on fracture healing, but also the obvious changes in the 3D network of the callus strongly indicate the impairment of bone remodelling.

With respect to prednisolone, the dose of 0.5 mg/kg body weight per day to generate human equivalent pharmacological data was taken from the literature [33]. To date, there is no clear evidence that the fracture healing is restricted in patients who are under glucocorticoid medication [34]. Controversially, long-term medication of 5.5 weeks up to 3.5 months led to impaired fracture healing in rabbits (ulnar osteotomy) and rats (closed/open fracture of the femur) [7, 10, 19, 33–35], whereas short-term medication of 3-4 days in a femural osteotomy model of the rat did not affect the biomechanics or histology [16, 36]. Here, the applied doses ranged from 0.02 mg/kg to 2 mg/kg body weight per day.

The data from our prednisolone group revealed no relevant difference in the elevated BV in comparison with that of the control group. However, the value was significantly higher than that of the diclofenac group. Concordantly, the prednisolone group of a recent rat femur fracture study showed an increased total bone area histologically generated by an increase of the woven bone, whereas the lamellar bone showed decreased values [34]. Compared with the control group, the authors described a slow remodelling of woven bone to lamellar bone within the callus; this is equivalent to a delay of fracture healing. However, since the authors did not undertake biomechanics or 3D tomography, no clear evidence could be drawn with regard to functional incompetence. Weinstein et al. have demonstrated decreased osteoclast production, resulting in a reduction of bone remodelling [8].

Thus, in contrast to NSAIDs the effect of steroids on fracture healing seems to be more complex. Via inhibition of phospholipase A2, prednisolone acts in the same pathway as diclofenac does, finally reducing prostaglandins locally. Additionally, a direct transcriptional downregulation of osteoprotegerin (OPG) occurs in osteoblast linages, independent of their respective differentiation [37, 38], whereas RANKL (Receptor activator of nuclear factor (NF-) kB ligand) is upregulated [39]. Recently, a study by Pichler et al. showed an increased RANKL expression in rats with prednisolone-induced osteoporosis leading to an enhanced RANKL/OPG ratio [40]. Interestingly, these effects could be reversed by physical activity leading to protective effects not only on bone, but also on articular cartilage [41].

In accordance, our histological results revealed bony fracture gap bridging in only 17 % of the prednisolone group (control group: 33 %). In 50 % of the specimens, fibrous connective tissue in the area of the fracture gap (control group: 33 %) was still present, whereas cartilage was detected rarely compared with the control group. Gerstenfeld et al. described a transition from cartilage to bone after 21 days in physiological fracture healing in the rat and, similarly, the residual presence of connective tissue in our prednisolone group can be interpreted as a delay of fracture healing [42]. With regard to the structural parameters assessed by μCT in our study, the SMI of the prednisolone group was significantly lower (-1.30) than the SMI of the diclofenac group and a trend was shown compared with the control group. Negative values represent dense stable trabecular structures. The Tb. Th. was significantly higher compared with the diclofenac group implying a stronger network. Thus, unlike diclofenac, the prednisolone-influenced callus seemed to be more stable, thereby epitomising the delayed remodelling via osteoclast inhibition. Nevertheless, simultaneous osteoblast inhibition resulted in an impairment of the bridging callus, together resulting in lower union rates. Thus, biomechanically, a markedly decreased breaking load and stiffness of the prednisolone group was shown compared with the control group. Histologically, the alterations were confirmed: in spite of an adequate callus volume, even the outer periosteal mineralised callus did not bridge the gap in most of the cases. However, this is the area in which cortical bridging normally begins and, since it is located far from the geometric centre of the bone, it is considered to be responsible for the majority of weight bearing [14, 29].

## Conclusion

Summarised, diclofenac and prednisolone showed substantial impairment of fracture healing in the rat fracture model. Therefore, in particular for patients with risk factors (diabetes mellitus, smoking), these drugs should be avoided when fracture healing or spine fusion is the purpose of treatment. Because the administration of prednisolone results in impaired delayed fracture healing in a different way from that of NSAR application, further basic research is needed to determine the causes for this and, thus, to develop new causal therapy approaches. However, findings in animal studies cannot be extrapolated directly to humans and so studies are necessary to investigate the possible (side)-effects of these medications in patients [43]. Furthermore, an important aspect would be to determine the cut-off duration for the “safe” administration of diclofenac and prednisolon in the clinical setting of fracture healing.

## Additional file

Additional file 1: μCT diclo, μCt pred, μCT control, biomech diclo, biomech pred, biomech control. Description of data: single values, mean and median. (XLSX 42 kb)

## Abbreviations

%: Percentage
3D: 3-dimensional
AB: Air bubble
BM: Bone marrow
BMC: Bone mineral content
BS: Bone surface
BV: Bone volume
CA: Cartilage
CB: Original cortical bone
CC: Calcified cartilage
COX-1 and-2: Cyclooxygenase isoenzymes 1 and 2
DA: Degree of anisotropy
F: Fracture site
FCT: Fibrous connective tissue
i.m.: Intramuscular
LL: Laczkó and Lévai
mean ± SD: Mean values ± standard deviation
MMA: Methyl methacrylate
NSAIDs: Non-steroidal anti-inflammatory drugs
OC: Osseus callus
OPG: Osteoprotegerin
PGs: Prostaglandins
RANKL: Receptor activator of nuclear factor (NF-) kB ligand
s.c.: Subcutaneously
SMI: Structure model index
Tb. Th.: Trabecular thickness
TMD: Tissue mineral density
VOI: Volume of interest
WB: Woven bone
μCT: Micro-CT
